# OrthoList: A Compendium of *C. elegans* Genes with Human Orthologs

**DOI:** 10.1371/journal.pone.0020085

**Published:** 2011-05-25

**Authors:** Daniel D. Shaye, Iva Greenwald

**Affiliations:** 1 Howard Hughes Medical Institute, Columbia University, College of Physicians and Surgeons, New York, New York, United States of America; 2 Department of Biochemistry and Molecular Biophysics, Columbia University, College of Physicians and Surgeons, New York, New York, United States of America; 3 Department of Genetics and Development, Columbia University, College of Physicians and Surgeons, New York, New York, United States of America; Thomas Jefferson University, United States of America

## Abstract

**Background:**

*C. elegans* is an important model for genetic studies relevant to human biology and disease. We sought to assess the orthology between *C. elegans* and human genes to understand better the relationship between their genomes and to generate a compelling list of candidates to streamline RNAi-based screens in this model.

**Results:**

We performed a meta-analysis of results from four orthology prediction programs and generated a compendium, “OrthoList”, containing 7,663 *C. elegans* protein-coding genes. Various assessments indicate that OrthoList has extensive coverage with low false-positive and false-negative rates. Part of this evaluation examined the conservation of components of the receptor tyrosine kinase, Notch, Wnt, TGF-ß and insulin signaling pathways, and led us to update compendia of conserved *C. elegans* kinases, nuclear hormone receptors, F-box proteins, and transcription factors. Comparison with two published genome-wide RNAi screens indicated that virtually all of the conserved hits would have been obtained had just the OrthoList set (∼38% of the genome) been targeted. We compiled Ortholist by InterPro domains and Gene Ontology annotation, making it easy to identify *C. elegans* orthologs of human disease genes for potential functional analysis.

**Conclusions:**

We anticipate that OrthoList will be of considerable utility to *C. elegans* researchers for streamlining RNAi screens, by focusing on genes with apparent human orthologs, thus reducing screening effort by ∼60%. Moreover, we find that OrthoList provides a useful basis for annotating orthology and reveals more *C. elegans* orthologs of human genes in various functional groups, such as transcription factors, than previously described.

## Introduction

*C. elegans* has been an important model for elucidating conserved pathways and processes relevant to human biology and disease. There are between ∼20,250 and ∼21,700 predicted protein-coding genes in *C. elegans* (WormBase referential freeze WS210, Jan. 2010 and [1]). It is clear that many of these genes are shared with humans, but also that many are not. However, there has been no definitive study of the relationship between the two genomes. We have undertaken the assessment presented here to better understand the relationship between the genome of this useful model organism and the human genome, with the practical goal of identifying a compelling list of orthologs that can be used to streamline functional genomic screening.

The prediction of genes that most likely share a function between species generally makes use of evolutionary relationships inferred from sequence analysis. Genes in two species that directly evolved from the same gene in their last common ancestor are more likely to have a conserved function. Such genes are called “orthologs” [2] and they are typically identified, using a basic local alignment search tool (BLAST) [3] search, as the reciprocally-best hits (RBHs) in both genomes. However, a high degree of gene duplication, particularly in distantly related organisms, hinders ortholog identification by the RBH approach, as this approach only identifies the best hit, and not all the gene copies that may retain some common function. Genes that arise by duplication are termed “paralogs” [2], and two kinds of paralogs have been recognized [4]: “in-paralogs” (also called “co-orthologs”, see [5]), for which the duplication of the ancestral gene occurred after speciation, and “out-paralogs”, for which the duplication event occurred prior to speciation. Even though orthology and paralogy are, strictly speaking, phylogenetic definitions, for the purposes of inferring function from these evolutionary relationships, it is typically assumed that orthologs, and to some extent in-paralogs, retain similar functions [4], [5]. Indeed, for several *C. elegans*-human orthologs the idea that these proteins have retained function has been experimentally verified (reviewed in [6]). Therefore, our goal for this analysis was to compile a reasonable list of orthologs and in-paralogs by comparing the *C. elegans* and human genomes.

In addition to interest as a problem in genome sequence analysis, there is an important practical reason for defining genes conserved between humans and *C. elegans*: facilitating double-stranded RNA mediated interference (RNAi)-based forward genetic screens. The discovery of RNAi [7] revolutionized functional genomic analysis. The ability to perform RNAi by feeding worms bacteria expressing double-stranded RNA for individual genes [8] led to the generation of a “feeding library” that covers many of the predicted protein-coding genes in the genome [9], [10], facilitating gene discovery efforts. Although screens may be done on a genome-wide scale using the entire feeding library, RNAi also offers the possibility of selectively targeting particular subsets of genes based on specific criteria to streamline gene discovery efforts (for an example, see [11]). Identifying a subset of *C. elegans* genes with human orthologs would be especially useful for gene discovery with translational potential to human disease, because the effort spent on screening and analysis could be cut significantly by eliminating genes for which counterparts in humans do not exist or cannot be recognized by primary sequence data.

The available comparisons of the *C. elegans* and human genomes are out of date and suffer from a reliance on early or incomplete drafts of genome sequence information, as well as on earlier, less powerful sequence analysis methods. These problems have led to greatly disparate results, predicting anywhere from ∼1,800 to ∼15,000 *C. elegans* genes as having human orthologs [6], [12]–[15]. As all sequence analysis methods have advantages and disadvantages [16]–[18], we decided to adopt the approach of a meta-analysis of results from four different orthology-prediction programs to compile an authoritative list of genes. Details of the strategy are described here. Our analysis has yielded a set of 7,663 genes that should be useful for RNAi screens with translational potential and are presented in formats that are easily accessible for the purposes of designing screens and readily identifying *C. elegans* and human gene counterparts.

## Results and Discussion

### Rationale for a meta-analysis

There is currently no “gold standard” for identifying a complete set of orthologous genes between two species. The easiest and most widely used approach is to perform BLAST searches to define the RBHs in both genomes. However, this approach is not particularly sensitive: it only reports the best counterpart in each genome, thus missing any paralogs that may be functionally relevant. Indeed, it has been suggested that the RBH approach may fall prey to a 30 to 40% false-negative rate (depending on how “best-hit” is defined) [17] when performing large-scale ortholog identification. We also found that the RBH criterion failed to identify several validated human-*C. elegans* orthologs (for examples see our discussion of the transcription factor compendium below).

Several automated programs for genome-wide assignment of genes into orthologous groups (which include orthologs and in-paralogs) have been developed in order to address the shortcomings of the simple RBH approach (reviewed in [16]–[18]). All such programs initially assign orthology based on BLAST searches, but then differ on how they deal with paralogs. Broadly speaking, the methods can be classified into two categories: those that group genes based on primary sequence comparisons, and those that do so by generating sequence similarity trees and reconciling them to phylogenetic species trees. Both approaches have advantages and disadvantages [16]–[18], suggesting that a meta-analysis that includes programs incorporating both approaches might give the most accurate picture of how the *C. elegans* and human genomes compare.

One possible problem with such automated genome-wide methods that has been raised [15] is that they are dependent on the quality of gene prediction in the genomes under scrutiny, which can sometimes be ambiguous or outdated. This potential issue for any genome-wide analysis does not in itself preclude the utility of such an approach, but it should be kept in mind that orthology predictions made on a single and/or outdated data set is likely not to be entirely accurate. A meta-analysis of various orthology prediction methods that use recent releases of the reference genomes (see Table 1) addresses this concern for the present, even though it should be reassessed in the future (discussed further in the “Conclusions” section).

**Table 1:** *C. elegans* and human genome releases underlying orthology prediction programs.

Orthology Database	KOG	TreeFam	InParanoid	OrthoMCL	HomoloGene	Ensembl Compara
(Version, Year)	(2003)	(v7, 2009)	(v7, 2009)	(v4)	(v64, 2009)	(v57, 2010)
WormBase release	WS67	WS190	WS199	WS199	WS190	WS200
(Date)	(2001)	(May 2008)	(Feb. 2009)	(Feb. 2009)	(May 2008)	(March 2009)
Protein-coding genes in WormBase	(20,275)^a^	20,177	20,178	20,178	20,177	20,168
Human genome assembly release	NCBI30	Ensembl50/NCBI36	Ensembl52/NCBI36	Ensembl53/NCBI36	NCBI37.1	Ensembl57/GRCh37
(Date)	(2002)	(Jul. 2008)	(Dec. 2008)	(Mar. 2009)	(Aug. 2009)	(Jul. 2009)
Protein-coding genes in assembly	(37,840)^a^	20,067	21,673	21,343	22,165	22,253

KOG and TreeFam [22], [23] were not included in the meta-analysis used to generate OrthoList. KOG is shown for historical perspective, while TreeFam was used to confirm, or refute, orthology assignments. InParanoid, OrthoMCL, HomoloGene and Ensembl Compara [4], [19]–[21] were used to generate OrthoList.

a For the KOG database, the number of reported proteins analyzed includes alternatively-spliced forms derived from a single gene. For the other methods typically a single (the longest) isoform was used in the analysis.

### Compiling a list of *C. elegans*-human orthologs

The four programs included in our meta-analysis (InParanoid, OrthoMCL, HomoloGene and Ensembl Compara) are rated highly by publications analyzing the performance of orthology-prediction methods [16]–[18] and are described in the “Materials and Methods” section (see also [4], [19]–[21]). We note that we did not include two widely referenced programs, the KOG database and Treefam. The KOG database is one of the first large-scale orthology databases for eukaryotes [22], but depends on manual curation and is not easily updated. The most recent update was done in 2003, so we excluded it from our analysis. TreeFam is a curated phylogenetic tree for gene families [23]. We excluded it because extracting genome-scale data from this database is very difficult, its performance was not assessed by the publications mentioned above, and the *C. elegans* genome reference sequence used in the currently-available TreeFam release is more outdated than that used by the methods we did analyze (see Table 1). We note that some of the methodology underlying TreeFam has been adapted for use in one of the programs we do query (Ensembl Compara, see below), and that we do use TreeFam to assess the hits obtained by, or missing from, the results of our meta-analysis (see below).

**Figure 1:**
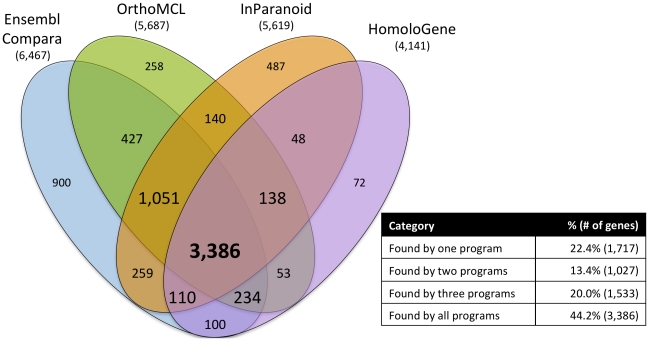
Comparison of four orthology prediction programs queried for *C. elegans* orthologs of human proteins. This diagram is modified from VENNY (see Materials and Methods). Each program is named above the oval representing its results, with the number of *C. elegans* orthologs and in-paralogs found by the program shown. The table gives an overall measure of how many genes were found by one or more programs (regardless of which one(s) found them). The numbers in the overlapping and non-overlapping areas of the Venn diagram indicate how many genes were found by overlapping or unique sets of programs. The font size used for these numbers indicate how many programs that number of genes was found by: numbers corresponding to genes found by a single program are shown smallest, whereas the largest font denotes the number of genes found by all programs. The data underlying this diagram can be seen in Table S1. A measure of the similarity and divergence between programs can be found in Table S2.

When assayed for *C. elegans*-human orthologs, the four methods analyzed yielded different and overlapping results (see Figure 1 and Tables S1, S2). Comparison of these results (see Materials and Methods) resulted in a list of 7,663 unique protein-coding genes, which we call OrthoList. This list represents ∼38% of the 20,250 protein-coding genes predicted in *C. elegans* (WormBase referential release WS210).

We note that although the largest class in OrthoList was that of genes found by all four methods (3,386 genes; see Figure 1 and Table S1B), this class represents less than half (∼44%) of all genes in the list. However, assigning orthology by restricting OrthoList to just this class of genes would be too restrictive, because there are many genes scored as orthologs by a single method (most often Ensembl Compara, see Table S2A) that, as we will show below, are known to be functionally relevant. Thus we included genes found by even a single method as a conservative approach to help ensure that most genes with human orthologs and/or conserved function are represented in OrthoList.

Finally, we also note that the distribution of *C. elegans* orthologs predicted by these programs follows independent performance assessments of these distinct methods. For example, we find that HomoloGene predicted the smallest number of orthologs, but this prediction is highly congruent with the other methods: ∼98% of orthologs predicted by HomoloGene are found by at least one other method (see Figure 1 and Table S2B), which may suggest a low false-positive rate for HomoloGene. This observation is consistent with the idea that this program performed best (i.e. was more “specific” or had fewer false-positives) in both phylogenetic and functional tests in a recent assessment of orthology-prediction methods [16]. However, in that same assessment it was pointed out that HomoloGene had relatively low coverage compared to other methods, and that if one wishes to increase “sensitivity” (i.e., reduce the false-negative rate), OrthoMCL and InParanoid offered increased coverage at a somewhat lowered specificity. At the other extreme, the same assessment found that, of the methods used here, Ensembl Compara was the least specific. Indeed, we find that this method was the least congruent with the others and provided the most unique hits (see Figure 1 and Table S2) which may suggest a higher false-positive rate. However, given that in many contexts (particularly when selecting genes for functional RNAi screens) it would be preferable to increase coverage at the expense of including some false-positives, we believe that the increased sensitivity, at the expense of reduced specificity, afforded by including genes predicted even by a single method is a worthwhile tradeoff.

### Assessing the specificity and sensitivity of OrthoList

We assessed the rate of false-positives and false-negatives in OrthoList by comparing it to three manually curated lists of predicted *C. elegans*-mammalian orthologs: protein kinases, nuclear hormone receptors (NHRs) and F-box proteins [24]–[27]. These lists represent large and well-studied families that exhibit different degrees of conservation outside nematodes, as well as different levels of nematode-specific expansion and divergence.

We note that the methods used to find orthologous family members in these previously curated lists are largely independent of those used by the programs we analyzed to compile OrthoList (see Materials and Methods). Thus, comparing the genome-wide approach of OrthoList to these independently compiled lists of specific families should allow us to assess how well the programs used to construct OrthoList are able to recognize orthologs and in-paralogs. For this comparison we extracted protein kinases NHRs and F-box proteins from OrthoList (see Materials and Methods), and found false-negative rates of 6 to 14% and false-positive rates of 6 to 9% with respect to previous compendia of these families (see below). This analysis suggests that, combined, the four programs we used to generate OrthoList are both sensitive and specific at detecting *C. elegans* orthologs of human proteins.

#### Protein kinases

**Figure 2:**
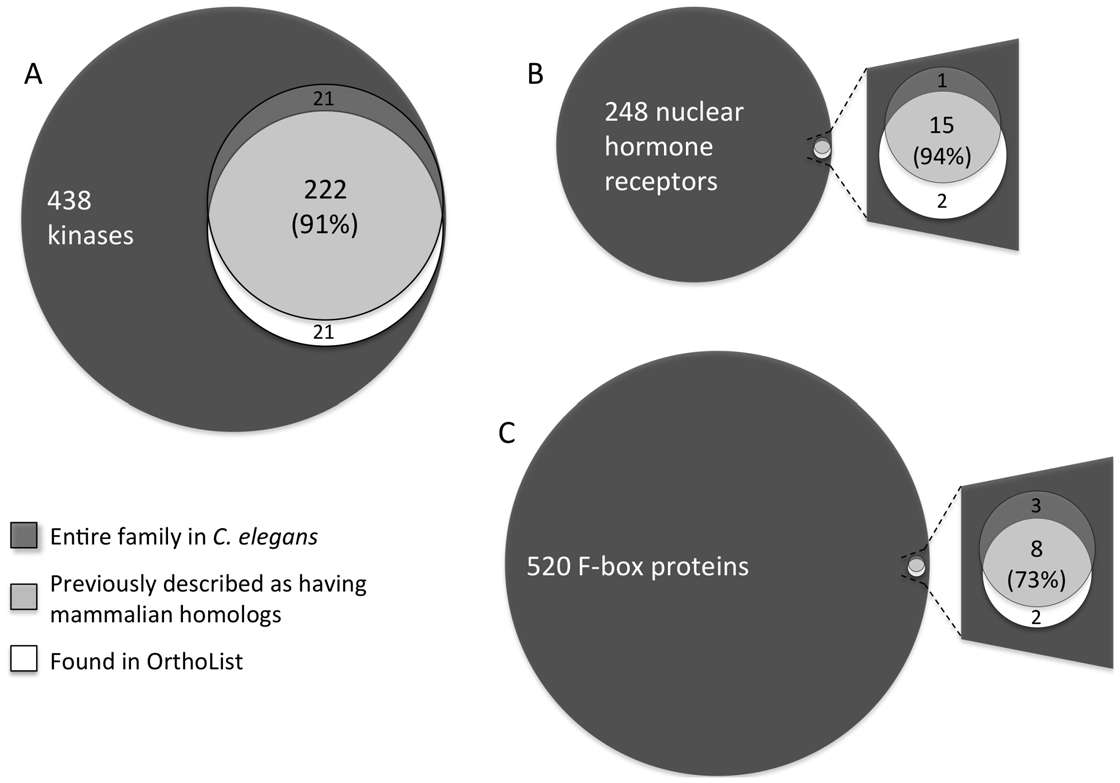
Examining OrthoList specificity and sensitivity. Venn diagrams comparing *C. elegans* gene families, and their previously defined conserved subsets, to members of the same families found in OrthoList (see Materials and Methods). For each family the overlap between OrthoList and the previously defined conserved subset is shown (the percentage refers to how well covered by OrthoList the conserved subset is). The possible homologs missing from OrthoList (putative false-negatives, shown above each overlap) and those found in OrthoList not previously defined as homologs (putative false-positives, shown below each overlap) are based on homology assignments of the original compendia for each of these families (see Materials and Methods, and [24]–[28], [56]). As discussed in the main text, the number of false-negatives and false-positives may actually be lower. A) Kinases (see Table S3C for source data). B) NHRs (see Table S4A for source data). C) F-box proteins (see Table S4B for source data).

The most recent available survey suggested that the *C. elegans* genome encodes 438 predicted protein kinases, and that almost half of them are members of worm-specific or worm-expanded families [26]. We manually updated this previously published list of kinases (see Materials and Methods, and Table S3A) and compared it to OrthoList. We found that OrthoList contains 243 kinases (Table S3B), which represents ∼55% of the predicted *C. elegans* kinome (see Figure 2A). Importantly, the OrthoList kinome is highly congruent with the previously predicted conserved kinases: the initial analysis suggested that 222 out of 243 (∼91%) kinases previously described as having mammalian orthologs are found in OrthoList (see Figure 2A and Table S3C), so that the false-negative rate for OrthoList for kinases is no more than 9%. However, further analysis suggests that it may be as low as 1%, because most (19 out of 21) of the kinases previously thought to have mammalian homologs are either not currently grouped with human kinases, or are predicted to be out-paralogs, by TreeFam (see Table S3D).

OrthoList also shows a low false-positive rate with respect to kinases: only 21 (∼9%) of the kinases in our list were not previously thought to have human homologs (see Figure 2A and Tables S3C, E). Most of these may really be false-positives, as TreeFam groups them either in nematode-specific trees or as nematode-specific out-paralogs in more conserved trees (see Table S3E). However, four kinases in this group may not be false positives. Two (*C24A8.4/cst-2* and *C27D6.11*) were picked by all four of the orthology-prediction programs used to build OrthoList and are, in addition, scored by TreeFam as having human orthologs. The other two (*R02C2.6* and *Y4C6A.1*) appear in TreeFam as in-paralogs of *C. elegans* kinases with human orthologs. Thus, these four kinases may be true positives not previously thought to have human orthologs (see Table S3E).

#### Nuclear hormone receptors

NHRs have undergone immense expansion in nematodes: there are 248 predicted NHRs in *C. elegans* (compared to 48 in humans), and of these only 16 (or ∼6%) were previously defined as being conserved between *C. elegans* and humans (see Table S4A and [24], [25]). When we checked OrthoList, we found that it contains 17 NHRs, of which 15 correspond to those previously described as having human orthologs (see Figure 2B and Table S4A). One NHR previously described as being conserved (*nhr-48*) is missing from OrthoList, while two not previously described as conserved (*nhr-14* and *nhr-35*) are on the list, both found by a single method (Ensembl Compara). This initial analysis suggested that, for NHRs, OrthoList has a false-negative rate of ∼6% (1 out of 16 NHRs not picked up in OrthoList) and a false-positive rate of ∼12% (2 out of 17 NHRs in OrthoList not previously thought to have human orthologs). However, upon further inspection, we believe that one of these two putative false-positives may be a true positive, as *nhr-35* is also predicted by TreeFam to be an in-paralog of *nhr-49*, *nhr-64* and *nhr-69* (see Table S4A), all of which are described as having human orthologs [24], [25].

#### F-box-containing proteins

The F-box family has also undergone tremendous expansion in nematodes. There are ∼520 predicted F-box protein-coding genes in *C. elegans*, whereas there are only 68 in humans [27], [28]. Much of this expansion is postulated to be due to adaptive evolution in response to host-pathogen arms races: it was proposed that the SCF1 ubiquitin-ligase complex, which uses the F-box-subunit as its target-recognition module, has been co-opted for binding and degradation of *C. elegans* pathogens [27]. As such, most of the *C. elegans* F-box genes are phylogenetically unstable (i.e., not conserved even among related nematodes) and seem to be rapidly evolving [27]. Estimates of conservation between *C. elegans* and mammals vary from 7 to 11 of the 520 (∼1–2%) *C. elegans* F-box proteins being conserved (see Table S4B and [27], [28]).

We found 10 F-box protein-coding genes in OrthoList (see Materials and Methods), which includes 6 of the 7 predicted by Jin et al. [28] and 8 of the 11 suggested by Thomas [27] (see also Table S4B and Materials and Methods). This initial analysis suggested a false-negative rate, with respect to this family, of ∼14–27%. However, two of the three F-box genes not in OrthoList (*T28B11.1 and T28B4.1*) also do not have mammalian orthologs in TreeFam (see Table S4B), suggesting that they may not be true positives. Removing them from the reference list would bring down the OrthoList false-negative rate to ∼12–14%.

The false-positive rate for F-box genes is surprisingly low, considering the large size of this family, with only two (*C10E2.2* and *Y60A3A.8*) not previously predicted to have mammalian orthologs found in the OrthoList. Indeed, *C10E2.2* may not be a false-positive, as it is found up by two different orthology-prediction programs (Ensembl Compara and InParanoid) and it is grouped as the ortholog of mammalian FBXO30/FBXO40 by TreeFam. Moreover, RNAi against *C10E2.2* showed that it might play a role in muscle development in *C. elegans* [29], suggesting it has an endogenous target or targets. This is in contrast to the expanded and diverged class of F-box proteins, which likely target exogenous pathogens and which mostly do not show any phenotypes by RNAi [10], [27]. Taken together, these observations suggest that *C10E2.2* may not represent a false-positive, but may indeed be a newly described ortholog of human F-box proteins.

### OrthoList coverage of conserved signaling pathways

**Figure 3:**
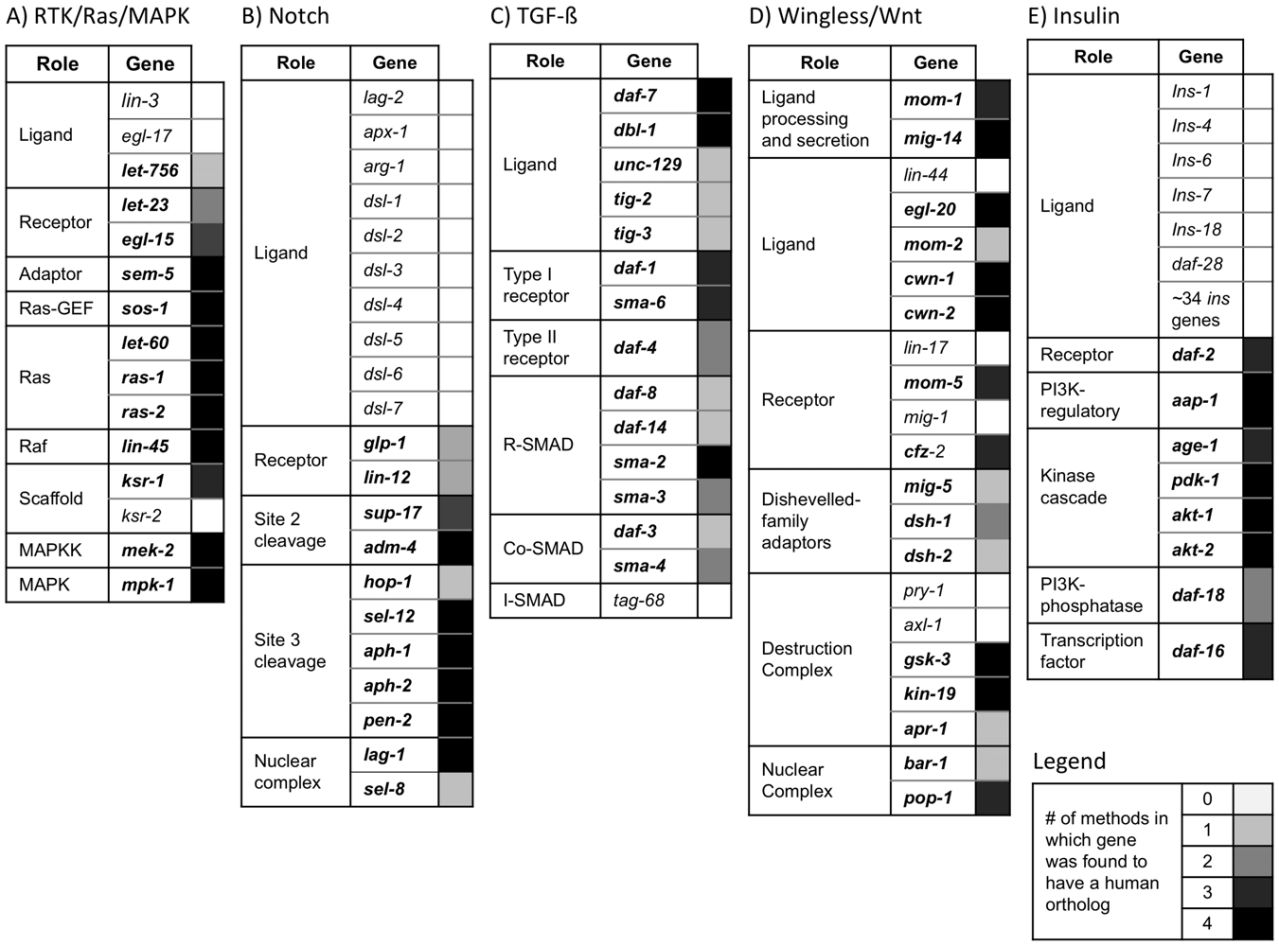
OrthoList coverage of conserved signaling pathways. Genes in bold are found by at least one orthology-predicting program, and thus included in OrthoList. The source data for this figure can be found in Table S5. A) RTK/Ras/MAPK pathway (reviewed in [34]). Note that *ras-1* and *ras-2* have not been defined functionally, although they are highly conserved. B) Notch pathway (reviewed in reviewed in [32]). C) TGF-ß pathway (reviewed in [33]). We note that *tag-68*, was only defined by conservation and no phenotype has been associated with its loss. D) Wnt pathway (reviewed in [30]). Note that our analysis was restricted to the conserved, canonical Wnt pathway. E) Insulin pathway. We specifically highlight the six insulins (*daf-28*, *ins-1*, *ins-4*, *ins-6*, *ins-*7 and *ins-*8), out of forty, that have been found (by overexpression, biochemical methods, RNAi or by existence of a semi-dominant allele) to be functional (reviewed in [31]).

To evaluate further the utility and completeness of OrthoList we asked which components of known conserved signaling pathways are found in this list. To this end, we looked for members of the Receptor Tyrosine Kinase (RTK)/Ras/Mitogen-Activated Protein Kinase (MAPK), Notch, Wingless (Wnt), Transforming Growth Factor Beta (TGF-ß) and Insulin pathways (reviewed in [30]–[34]). We found that for these known conserved pathways most of their core components, particularly those involved in signal transduction, are found in the OrthoList (see Figure 3 and Table S5). Thus, we believe that an RNAi library based on this list should reduce the function of these pathways, and by extension of other conserved pathways, at one or more steps. In addition, for each pathway, there are interesting observations regarding the nature and extent of the conservation between *C. elegans* and humans.

#### RTK/Ras/MAPK

Most components that function downstream of the receptors seem to be well conserved, as they are recognized by all four methods (the only exception being the partially redundant KSR scaffold proteins [34], of which only *ksr-1* is in OrthoList (found by three methods) while *ksr-2* is not (see Figure 3A and Table S5A). In addition, the known receptors bound by these ligands (*let-23/EGFR* and *egl-15/FGFR*) are found in OrthoList but not by all methods (*let-23* by only two and *egl-15* by three). In contrast, the known ligands are not particularly well conserved: *lin-3/EGF* is not found in OrthoList and neither is *egl-17/FGF* (although it is found to have a human ortholog by TreeFam), while *let-756/FGF* is found by a single method (Ensembl Compara).

#### Notch

Again, most of the downstream components of the Notch pathway are highly conserved and found by all orthology-prediction methods, the only exceptions being those that have partially redundant paralogs (i.e., *sup-17*, an ADAM protease, and *hop-1*, a presenilin). *sel-8*, a nuclear factor believed to be functionally equivalent to Mastermind but highly diverged in sequence (reviewed in [32]) is on the OrthoList (suggested by a single method, Ensembl Compara) as a possible ortholog of the Mediator complex subunit MDT15, raising the question of whether this protein may play a heretofore unknown role in Notch signaling in mammals and whether SEL-8 is truly the counterpart of Mastermind.

In contrast, none of the ten ligands predicted based on the presence of a DSL domain [35] are found in OrthoList (see Figure 3B and Table S5B), suggesting that the *C. elegans* ligands are very diverged. However, both of the Notch-family receptors, *lin-12* and *glp-1*, are found in OrthoList, but only by two methods, suggesting a degree of divergence in the receptors that is less extreme than the divergence of ligands.

#### TGF-ß signaling

All of the known, validated components of this pathway are in the OrthoList (see Figure 3C and Table S5C). The only potential component missing is *tag-68*, which resembles by sequence an inhibitory SMAD [36] but has not been assessed functionally for a role in TGF-ß signaling [33].

#### Wnt

In *C. elegans* there are canonical and non-canonical Wnt pathways, and the non-canonical one(s) are distinct from those considered non-canonical in vertebrates (reviewed in [30], [37]). We therefore focused our analysis only on components of the conserved canonical pathway.

Once again, we find that not all ligands or receptors, but most of the downstream components, are included in OrthoList. (see Figure 3D and Table S5D). The only downstream component not included is Axin, as the two *C. elegans* Axin homologs (*pry-1* and *axl-1*) are known to have greatly diverged in sequence [38], [39]. Importantly, two key components that function in Wnt production and secretion, *mom-1/*Porcupine and *mig-14/*Wntless (reviewed in [30], [37]), are found in the OrthoList. These upstream components are ideal targets for RNAi screens, as they are non-redundant genes whose loss can cause strong loss of Wnt signaling in *C. elegans* [40], bypassing the redundancy issues caused by having multiple ligands, receptors and downstream effectors (for an example see [41]).

#### Insulin

Sequence-base methods alone were previously shown to be insufficient to identify *C. elegans* insulin-like proteins, so a combination of sequence-based methods and stereochemical-restriction models was necessary to identify ∼40 genes encoding insulin-like proteins [31], [42]. Given this divergence, it is not surprising that none of these ligands are found in OrthoList. However, all of the other components, from the receptor *daf-2/InsR* to the most downstream transcription factor *daf-16/FoxO*, are in OrthoList (see Figure 3E and Table S5E)

In sum, key components of the signal transduction pathways queried are all found in OrthoList. Therefore, RNAi screens based on this list should reveal if conserved signaling pathways are involved in processes being analyzed.

### Comparing OrthoList and genome-wide RNAi screens

As an important goal of our analysis was to generate a streamlined list of clones for RNAi screening, we wanted to know how a screen carried out with the OrthoList subset of genes would compare to genome-wide screens in identifying conserved hits. To this end, we compared OrthoList to the results from two genome-wide RNAi screens that addressed fundamental conserved biological processes: cell division and endocytic/secretory trafficking [43], [44]. Our analysis suggests that virtually every gene identified in these screens that has a human ortholog would have been detected if only the much smaller OrthoList set had been used for the screen.

**Figure 4:**
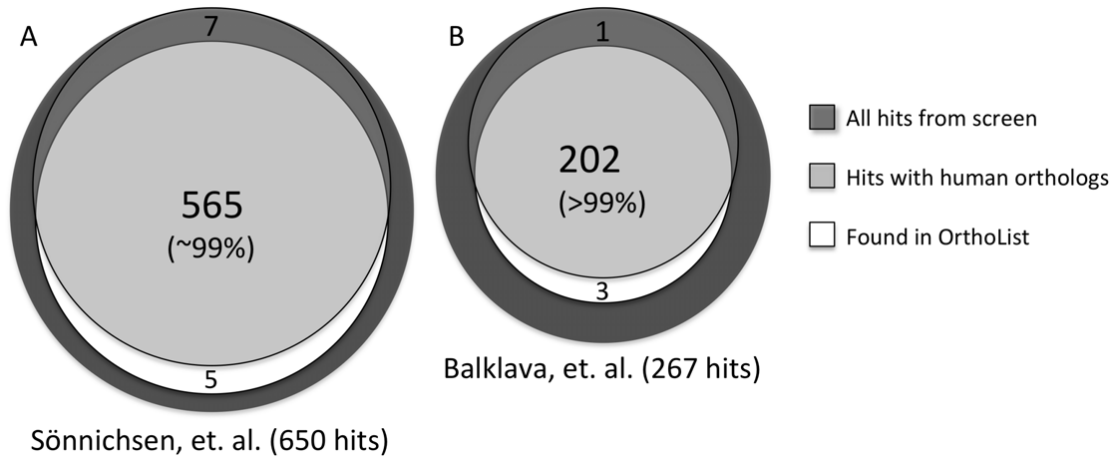
OrthoList coverage of hits from genome-wide RNAi screens. Venn diagrams analyzing hits obtained from RNAi screens examining (A) cell division [43] and (B) endocytic/secretory trafficking [44]. For each screen the overlap between OrthoList and the orthologous subset of hits is shown (percentage refers to how well covered by OrthoList this conserved subset is). Orthology assignments for hits missing from OrthoList (shown above overlap) and those found in OrthoList that were not called homologs in the original publications (shown below overlap) were confirmed by TreeFam and/or RBH (see Materials and Methods). Source data for these diagrams can be found in Tables S6 and S8.

In the cell division screen, Sönnichsen et al. used an injectable RNAi library targeting 19,075 genes and found that 661 affect the first two rounds of cell division during *C. elegans* embryogenesis [43]. Updating the list of hits (see Materials and Methods) shows that only 652 of these still exist in the most recent genome release (see Table S6A). To get a sense of what the results would be if only the genes from OrthoList had been used for the screen, we wanted to know how many of the hits with human homologs are in our compendium. Sönnichsen et al. suggested that 575 of their hits had human homologs (see [43] and Materials and Methods). However, our analysis, by RBH and TreeFam, suggests that at least 17 hits previously thought to have human homologs do not do so by these criteria (see Table S6D), while 14 hits previously not thought to have homologs actually do (see Table S6E). Thus, the actual number of hits from this screen with human orthologs is 572, of which 565 are in the OrthoList (see Figure 4A and Tables S6B, C). Therefore, had the cell division screen been done against just the OrthoList set, ∼99%, of the conserved hits would have been recovered performing only ∼40% of the injections, representing a significant savings in time and effort.

In the trafficking screen, Balklava et al. [44] used the widely available “feeding library” [10]. This library targets ∼16,300 genes, including 6,198 of the genes in OrthoList (representing 81% of this compendium. See Table S7). After screening the entire feeding library, Balklava et al. found 268 candidate trafficking regulators [44], of which 267 still exist in the current genome prediction (see Table S8A). Although it was previously suggested that 215 of the hits had human homologs (see Materials and Methods, and [44]), our analysis (by RBH and TreeFam) suggests that at least 20 hits previously thought to have human orthologs do not (see Table S8D), while 8 that were not thought to have human orthologs appear to do so (see Table S8E). Therefore, the actual number of hits from this screen with human orthologs is 203, of which 202 are in the OrthoList (Fig. 4B, Table S8B). Therefore, had the trafficking screen been carried out against just the OrthoList set, >99%, of the conserved hits would have been recovered with just ∼40% of the work.

We note that in both screens, several hits previously not thought to have human orthologs were found in OrthoList, and we confirmed their orthology by RBH and TreeFam (see Tables S6E, S8E). Defining hits with human homologs is an important aspect of RNAi screening, as it has implications in prioritizing further studies and applicability to humans. Thus, OrthoList not only provides a mean to reduce work, but also to annotate results from RNAi screens in an apparently more sensitive manner than BLAST queries. This additional use of OrthoList is explored further below.

### Using OrthoList for functional annotation

**Figure 5:**
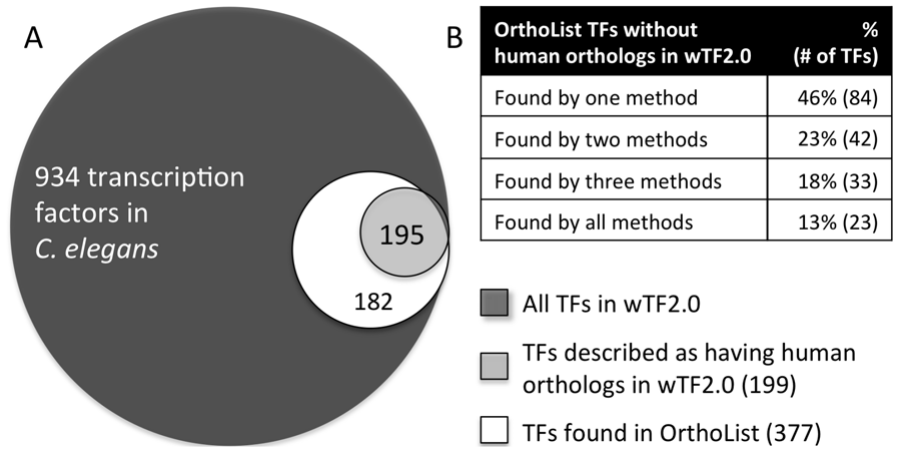
OrthoList coverage of a transcription factor compendium. A) Venn diagram comparing the wTF2.0 compendium [45] to OrthoList. Source data for this diagram is found in Tables S9A, B. We find that OrthoList contains ∼98% of TFs previously scored as having human orthologs (overlap). In addition, OrthoList contains 182 TFs not scored in wTF2.0 as having orthologs. B) Distribution of TFs found to have orthologs by OrthoList, but not by wTF2.0. Source data for this table is found in Tables S9C–E.

The annotation of human orthologs in *C. elegans* is potentially useful for functional genomic studies in this genetically tractable model organism. The underlying assumption that orthologs and in-paralogs often retain function [16]–[18] has been experimentally verified in *C. elegans* for some cases (reviewed in [6]). To evaluate the utility of OrthoList for functional annotation, we analyzed a previously compiled compendium of *C. elegans* transcription factors (TFs) called wTF2.0 [45]. Of the 934 TFs predicted by this analysis, only 199 were annotated, by RBH, as having human orthologs (see [45] and Figure 5A). We found that OrthoList predicts many more: 195 of the 199 (∼98%) TFs predicted in wTF2.0 to have human orthologs are found in OrthoList, and an additional 182 not annotated by wTF2.0 as having orthologs were also found (see Figure 5 and Table S9A).

This dramatic increase in putative orthologs raises the question as to whether the additional TFs found in our list are false-positives. We therefore asked whether those present in OrthoList, but not previously identified as having orthologs in wTF2.0, are supported by other criteria (e.g. previous publications and/or functional information). Few (∼13%) of the TFs annotated by OrthoList, and not by wTF2.0, were found by all the programs used to compile our list (See Fig. 5B and Tables S9C, D). Given that all methods pick them up, these are likely to truly have human orthologs, and indeed several of the genes in this class, such as *lin-39 (Hox4/5)*, *ttx-3 (Lhx2)* and *unc-62 (Meis)*, are considered to be orthologs of mammalian genes [46]–[48]. About 46% of TFs annotated by OrthoList, and not by wTF2.0, were found by a single method (most commonly Ensembl Compara; see Fig. 5B and Tables S9C, E). However, even within this class we found TFs previously considered to have human orthologs, such as the NHRs *daf-12* and *nhr-8* (co-orthologs of human *VDR*, *PXR* and *CAR*, see [24], [25]) and the factors *ceh-36* and *spr-1*, orthologous to the homeodomain gene *Otx* and the co-repressor *CoREST* respectively. Indeed, expression of mammalian *Otx* and *CoREST* in *C. elegans* has been shown to rescue *ceh-36* and *spr-1* mutants to some extent [49], [50], supporting the inference of orthology based on the low-level sequence homology. Taken together, these results suggest that OrthoList provides a sensitive way of assigning orthology that seems to be more robust than the simple RBH approach.

### Conclusions

We compiled OrthoList, a list of *C. elegans* orthologs of human genes, using four different orthology-prediction methods. By comparing to manually curated lists of large *C. elegans* gene families we have shown that OrthoList is very sensitive and specific at detecting orthologs. This list can provide a starting point for RNAi screens focused on conserved genes, which would greatly reduce workload. Indeed, we found that for two genome-wide screens, virtually all of the hits with predicted human homologs would have been found had just the OrthoList set been screened.

The efficacy of genome-wide orthology prediction approaches depends on the accuracy of the gene models in the genomes under scrutiny ([15] and see also discussion above). Genome annotation in both *C. elegans* and humans is an ongoing process that relies on various approaches. For example, a recent shotgun proteomics study in *C. elegans* [51] led to isolation of peptides encompassing 6,779 proteins, ∼94% of which were predicted in the then extant WormBase release (WS150. October, 2005). Interestingly, of the ∼6% of proteins that were erroneously predicted, or missing entirely, several were quickly and independently corrected (based on expression, homology and other data) by WormBase, even as the proteomic analysis was being prepared for publication [51]. These observations suggest that a majority of the gene models in the WS150 WormBase release were correct, and that there is continuous curation of these models. More recently, the *C. elegans* transcriptome profiling effort modENCODE [1], added new genes and changed some gene models that were present in the WormBase WS170 (January, 2007) release. The methods queried for our meta-analysis used more recent releases of the genome sequence (WS190 to WS200, see Table 1), which may have incorporated some of the modENCODE information. Given the constant flux of genomic data, OrthoList is likely to require updating. However, most conserved sequences are already represented in the genome releases used by the orthology-prediction methods analyzed here, so we believe that it is unlikely to undergo major changes.

We find that OrthoList includes most, but not all, of the components of known conserved signaling pathways, and many essential components of the five major pathways we examined. The inclusion of so many conserved components suggests that most, if not all, conserved pathways and processes should be represented and targeted by RNAi screens based on our list. Thus, our compendium achieves the practical application we set forth in its design; namely, to generate a sensitive and specific list of *C. elegans* genes with human homologs to streamline functional forward genetic screens. We note that the RNAi “feeding library” of Kamath et al. [10] includes 6,198 of the 7,663 genes in the OrthoList (see Table S7); so about 80% of the orthologs we identified are already available for screens.

Finally, OrthoList is a more sensitive approach than the RBH BLAST approach to annotating *C. elegans* and human orthologs. To increase the utility of OrthoList, we have further annotated this compendium with InterPro domains [52] and Gene Ontology annotations [53]. To this end we generated “gene-focused” and “annotation-focused” pivot reports. Gene-focused reports (Tables S10A, S11A, S12A and S13A) allow users to easily view all domains/annotations for a gene of interest. Conversely, annotation-focused reports (see Tables S10B, S11B, S12B and S13B) group all genes that have common annotation(s)/domain(s). These reports should provide an easy way to find shared domains and predicted cellular, biological and molecular functions of *C. elegans* genes with human orthologs. Thus, this compendium will be a useful resource for *C. elegans* investigators wishing to streamline genome-wide approaches to genes of broad general interest, and for researchers in other fields wishing to easily find related proteins between humans and this genetically tractable model.

## Materials and Methods

### Source data for meta-analysis

Data from orthology-detection methods analyzed in this study are publicly available. A brief description of each method is given below. Default parameters were used in all cases.

#### InParanoid [4], [54]

In this program an orthologous group is originated by “seed” orthologs, defined as BLASTP RBHs between two species. A paralog more similar to the within-species seed than to any sequence in the converse species is added to the group as an in-paralog. A paralog less similar to the seed than to a sequence in the converse species is deemed an out-paralog and not added to the group. We downloaded data from InParanoid version 7 (http://inparanoid.sbc.su.se/cgi-bin/index.cgi).

#### OrthoMCL [19]

In this program proteins related by BLASTP are linked in a similarity graph, where the proteins are nodes and edges represent their relationships. Edges are weighted to correct for systematic differences in comparisons between species (e.g., differences that may be attributed to nucleotide composition bias) and to minimize the influence of the similarity among paralogs on overall clustering. Then a Markov clustering (MCL) algorithm is applied to the weighted similarity graph. MCL uses flow simulation and globally considers all relationships in the graph to generate clusters. This seems to provide a robust method for discarding diverged out-paralogs, distant orthologs mistakenly assigned based on weak reciprocal best hits, and sequences with different domain structures. We downloaded data from OrthoMCL-DB [55] version 4 (http://www.orthomcl.org/cgi-bin/OrthoMclWeb.cgi).

#### HomoloGene [21]

Although a detailed description of this method has not been published, it is described in their website (http://www.ncbi.nlm.nih.gov/homologene/). Briefly, proteins from different species are compared using BLASTP and then placed into groups using a tree built from sequence similarity to guide the process. More closely related organisms are matched up first and then less related organisms are added as the tree is traversed toward the root. Orthologous and paralogous relationships between genes are assessed from these trees. We downloaded data from HomoloGene version 64.

#### Ensembl Compara [20]

This program also relies on generating trees of gene families, based on protein and reverse-translated coding-sequence, and reconciling these to phylogenetic trees of species. We downloaded data from Ensembl version 57 (http://mar2010.archive.ensembl.org/index.html).

Once data was downloaded, we needed to standardize gene names, because each method utilized a different way of referring to genes (e.g. InParanoid uses Wormpep protein names, while OrthoMCL uses WormBase gene identifiers; see Tables S1C–F). To this end, we used Ensembl BioMart (http://uswest.ensembl.org/biomart/martview) to transform the contents of each list to WormBase sequence names (see Table S1A) We then compared lists using “VENNY” (an interactive tool for comparing lists with venn diagrams, found at http://bioinfogp.cnb.csic.es/tools/venny/index.html) to obtain the data detailed in Table S1B and shown graphically in Figure 1.

### Comparing OrthoList to other compendia

To assess specificity, sensitivity and coverage of OrthoList, we compared it to different datasets. Below we briefly describe how these sets were previously compiled and how we updated them, when necessary, to the WormBase WS210 gene models. In cases where there were discrepancies in orthology assignment between OrthoList and the compendium being analyzed, we further assessed orthology based on RBH (taking the *C. elegans* protein and performing an NCBI BLASTP search, using default parameters, on the human genome (at http://blast.ncbi.nlm.nih.gov/Blast.cgi) then taking the top human hit and performing a BLASTP search against the worm genome either at the NCBI website and/or at WormBase). We also used TreeFam release 7.0 (http://www.treefam.org/) to further scrutinize cases where orthology assignment differed between test sets and OrthoList.

We note that these previously compiled compendia of kinases, NHRs, F-box proteins (discussed here) and TFs (see below) were generated in a “domain-driven” manner, meaning that in all these cases inclusion in the compendium and/or orthology assignment was based on analyses of critical domains that define each family. This is different from the programs used to compile OrthoList, which carry out domain-blind, genome-wide, all-against-all, whole protein analyses. For this reason, we can assume that the specific compendia we compare to OrthoList were derived in an independent manner, and thus it is appropriate to compare them to OrthoList.

#### Kinases

A list of *C. elegans* kinases, assembled using profile-hidden markov models, PSI-BLAST and homology prediction [56], was downloaded (http://kinase.com/celegans/) and kinases marked as having mammalian homologs were extracted. Updates to this initial list were published by Manning [26]: in particular, 35 kinases that were either not present, or not thought to have mammalian homologs, in the original publication [56] were reassessed as having homologs by Manning [26]. Conversely, there were two kinases (*T17A3.1/ver-1* and *R107.4*) thought to have mammalian homologs in the original publication that were not listed as such in the latter (we found both of these in OrthoList). Finally, we corrected the sequence name for 33 kinases and removed 2 no longer considered kinases in WormBase. This resulted in a corrected compendium of 243 kinases with mammalian homologs. All corrections and updates can be seen in Table S3A.

To find kinases in OrthoList we first assigned InterPro and GO annotations to all the OrthoList genes using Ensembl BioMart (see Tables S10, S11, S12, S13). We then searched for genes with annotations associated with protein kinases (see Table S3B). This netted 250 putative kinases in OrthoList. We compared these to the updated kinase compendium and found 219 common genes, meaning that OrthoList may have 31 kinases not found in the corrected kinase compendium. We analyzed these more closely, by manual WormBase searches, and found that 10 of these were not kinases, even though they had InterPro and/or GO annotations associated with kinases (see Table S3B). This correction brought down the number of OrthoList-only kinases from 31 to 21. Conversely, of the 24 genes in the corrected kinase compendium not found in OrthoList, 3 were actually in our list, but not annotated as kinases by GO or InterPro (see Table S3B). This brought the number of common kinases up to 222, and those missing from OrthoList down to 21. The distribution of all kinases is summarized in Table S3C, and kinases for which orthology assignment was not congruent in both lists are analyzed in detail in Tables S3D, E.

#### NHRs

Orthology between humans and the expanded *C. elegans* NHR family had been previously assigned by performing phylogenetic analysis on their DNA-binding domains (reviewed in [25]). We generated a list of 16 conserved NHRs (see Table S4A) based on reviews of this family in *C. elegans* [24], [25]. We searched OrthoList for nuclear hormone receptors by looking for genes with InterPro annotations IPR001628 (“Zinc finger, nuclear hormone receptor-type”), IPR000536 (“Nuclear hormone receptor, ligand-binding, core”) and the GO molecular-function annotation GO:0003707 (“steroid hormone receptor activity”). This search yielded the 17 genes shown in Table S4A.

#### F-box domain family

Jin et al. [28] used a combination of BLAST analyses and phylogenetic tree constructions, based on the putative substrate interaction domains and the F-box motif, to find possible orthologs of mammalian F-box proteins in *C. elegans*. This analysis yielded 7 putative orthologs. Subsequently, Thomas [27] analyzed the evolution of the F-box family in *C. elegans* and found 12 proteins that had best BLASTP similarity to mouse proteins than to any of the ∼450 “unstable” F-box genes in *C. elegans* [27]. Although not strictly defined as having human orthologs, we included these in our analysis. The only one defined by Thomas [27] that we excluded was *ZK328.8/tbx-7*, which turns out to not be an F-box protein at all. Thus, in total, the compendium of F-box proteins previously classified as having mammalian homologs consists of 7 to 11 genes (see Table S4B). To find F-box proteins in OrthoList we searched for genes with InterPro domain IPR001810 (Cyclin-like F-box), which yielded 12 genes. However, 2 of these (*T08D2.2* and *Y50D7A.1*) did not have an obvious F-box, as assessed by WormBase or SMART [57]. Thus we limited our analysis to the 10 clear F-box containing proteins in OrthoList (see Table S4B).

#### EGFR/Ras/MAPK, Notch, TGF-ß, Wnt and Insulin pathway components

The *C. elegans* counterparts of proteins involved in these conserved pathways were determined from reviews of these signal transduction cascades [30]–[34]. Once the gene sequence names for these proteins were obtained from WormBase we searched for them in OrthoList. Details of this analysis are shown in Table S5.

### OrthoList coverage of genome-wide RNAi screens

#### Cell division screen

We downloaded a list of positive hits from this screen [43] and updated it (from WormBase WS100 to WS210). We found that of the 661 positives from the original screen, 12 genes had changed: 7 are now considered pseudogenes or have been completely removed, and 2 have been merged to genes that were independent hits in the screen (thus they represent duplicates). These 9 genes were removed from further analysis (see Table S6A). Of the remaining 652 hits, 575 (88%) were previously classified as having human homologs [43]. However, upon comparison to OrthoList, we reassessed this classification. We found that 570 of the hits were in OrthoList, including 19 genes not previously assigned human homologs ([43]; see Table S6B). Further analysis, by RBH and TreeFam, showed that 14 out of these 19 should be reclassified as having human orthologs (see Table S6E). Conversely, there were 24 hits classified as having homologs that we did not find in OrthoList ([43]; see Table S6C). Further analysis showed that 17 out of these 24 should be reclassified as not having human orthologs (see Table S6D). Therefore, the number of hits from this screen with human orthologs is actually 572 (575 from original classification+14 reclassified as having orthologs by RBH/TreeFam – 17 reclassified as not having orthologs by RBH/TreeFam). Of these, 565 (∼99%) are found in OrthoList (see Table S6B).

#### Mapping OrthoList genes to “feeding” library clones

For comparing OrthoList to the trafficking screen performed by Balklava et al. [44], we first needed to know how many of the genes in OrthoList are found in the RNAi feeding library used in the screen. To this end, we downloaded the most current “mapping” of feeding library clones to WormBase (ftp://caltech.wormbase.org/pub/annots/rnai/). This mapping compares the clones in the library to the most recent (in this case WS218) WormBase gene models. We found that 6,198 genes from OrthoList (∼81%) are represented in the feeding library. However, we note that 6 of these genes are considered pseudogenes in WormBase release WS218, and 11 genes are represented by clones found to be incorrect either in the source lab or by others (see Table S7).

#### Trafficking screen

We downloaded a list of positive hits from this screen [44] and updated it (from WormBase WS157 to WS210). We found that of the 268 positives from the original screen, only 2 genes had changed (see Table S8A). *F53B8.1* was merged to *vab-10/ZK1151.1*, which was found as a separate hit in the screen. Therefore, *F53B8.1* was removed from further analysis. The second changed hit, *Y113G7B.21*, was merged to *mdt-17/Y113G7B.18*. This gene would have been excluded by Balklava et al. from further analysis, due to its likely role in transcriptional regulation [44]. However, for simplicity, we consider it here as a positive from their screen. Of the corrected 267 hits, 215 (∼81%) were previously classified as having human homologs [44]. However, upon comparison to OrthoList, we again reassessed homology assignments. We found that 205 of the hits from this screen were in OrthoList, including 11 genes not previously found to have human homologs ([44]; see Table S8B). Further analysis by RBH and TreeFam showed that 8 of these 11 should be reclassified as having human orthologs (see Table S8E). Conversely, there were 21 hits previously classified as having homologs that we did not find in OrthoList ([44]; see Table S8C). Further analysis showed that 20 of these 21 should be reclassified as not having human orthologs (Table S8D). Therefore, the number of hits from this screen with human orthologs is actually 203 (215 from original classification+8 reclassified as having human orthologs – 20 reclassified as not having orthologs). Of these, 202 (>99%) are found in OrthoList (see Table S8B).

### Using OrthoList to annotate the wTF2.0 compendium

We downloaded the wTF2.0 transcription factor list, which was created based on GO, InterPro and other domain prediction methods to define *C. elegans* TFs [45]. We compared this compendium to OrthoList and found 377 TFs in our list, including one gene that had to be corrected (*T01C1.3*, which was superseded by merging to *T01C1.2*. See Table S9A). Most (195/199, or ∼98%) of the TFs classified in wTF2.0 as having human orthologs were found in OrthoList (see Table S9A). Of the 4 previously classified as having orthologs that were not found in OrthoList (see Table S9B), three did not exhibit consistent results by RBH and TreeFam, while one was a NHR not classified as being conserved by previous analyses of this family [24], [25]. We examined the 182 TFs assessed as having orthologs by OrthoList, but not by wTF2.0, and found that few were picked by all orthology-prediction methods, while most were picked by a single method (Fig. 5B, Table S9C, D, E). Regardless, even within this last category there are TFs that are functionally related to their human homologs (see main text for discussion).

## Supporting Information

Table S1 Compiling OrthoList. A) Lists of *C. elegans* genes found by the four different orthology detection programs. B) Compiled OrthoList and distribution of hits among the different orthology prediction programs. C) Ensembl Compara results (*C. elegans*-human orthologs). D) OrthoMCL results (*C. elegans* genes and the orthologous groups they belong to). E) InParanoid results (*C. elegans* and human proteins grouped by orthologous clusters). F) HomoloGene results (*C. elegans* and human genes grouped by orthologous clusters).
(XLS)

Table S2 OrthoList statistics. A) Number of unique hits found by each program. B) Congruence between programs (% hits shared).
(DOC)

Table S3 Analysis of kinases. A) Corrected kinase compendium. B) OrthoList kinases: GO and InterPro annotations used to find kinases, and corrections applied to arrive at final list. C) Summary of kinase distribution between corrected compendium and OrthoList. D) Analysis of kinases found in corrected compendium, but not in OrthoList. E) Analysis of kinases found in OrthoList, but not in corrected compendium.
(XLS)

Table S4 Analysis of NHRs and F-Box proteins. A) NHRs. B) F-Box proteins.
(XLS)

Table S5 Analysis conserved signal transduction pathways. A) RTK/Ras/MAPK. B) Notch. C) TGF-ß. D) Wnt. E) Insulin.
(XLS)

Table S6 Analysis of Sönnichsen et al cell division RNAi screen. A) Screen hits updated to Wormbase WS210. B) Hits in OrthoList. C) Hits not in OrthoList. D) Assessment of orthology, based on RBH/TreeFam, for hits not in OrthoList. E) Assessment of orthology based on RBH/TreeFam, for hits in OrthoList not reported by Sönnichsen, et al, as having human homologs.
(XLS)

Table S7 OrthoList genes with clones in Kamath, et al RNAi feeding library.
(XLS)

Table S8 Analysis of Balklava et al trafficking RNA screen. A) Screen hits updated to Wormbase WS210. B) Hits in OrthoList. C) Hits not in OrthoList. D) Assessment of orthology, based on RBH/TreeFam, for hits not in OrthoList. E) Assessment of orthology based on RBH/TreeFam, for hits in OrthoList not reported by Balklava et al as having human homologs.
(XLS)

Table S9 Analysis of the worm transcription factor compendium, wTF2.0. A) wTF2.0 transcription factors found in OrthoList. B) wTF2.0 transcriptions factors not found in OrthoList. C) Distribution (by program) of TFs found in OrthoList, not scored as having human orthologs in wTF2.0. D) TFs found by all four programs used to compile OrthoList methods, but not scored as having human orthologs in wTF2.0. E) TFs found by single OrthoList programs, not scored as having human orthologs by wTF2.0.
(XLS)

Table S10 InterPro annotation of OrthoList. A) Gene-focused pivot report. B) InterPro domain-focused pivot report. C) All OrthoList genes with InterPro domains (6,951/7,633, or ∼91%), and their associated annotations. This is source data for pivot reports in Tables S10A, B.
(XLS)

Table S11 Biological process (bp) GO annotations of OrthoList. A) Gene-focused pivot report. B) bp-focused pivot report. C) All OrthoList genes with bp GO annotations (6,951/7,633, or ∼77%), and their associated annotations. This is source data for pivot reports in Tables S11A, B.
(XLS)

Table S12 Cellular component (cc) GO annotations of OrthoList. A) Gene-focused pivot report. B) cc-focused pivot report. C) All OrthoList genes with cc GO annotations (4,313/7,633, or ∼56%), and their associated annotations. This is source data for pivot reports in Tables S12A, B.
(XLS)

Table S13 Molecular function (mf) GO annotations of OrthoList. A) Gene-focused pivot report. B) mf-focused pivot report. C) All OrthoList genes with mf GO annotations (5,269/7,633, or ∼69%), and their associated annotations. This is source data for pivot reports in Tables S13A, B.
(XLS)
