# Correction: OrthoList: A Compendium of *C. elegans* Genes with Human Orthologs

**DOI:** 10.1371/annotation/f5ffb738-a176-4a43-b0e0-249cdea45fe0

**Published:** 2014-01-10

**Authors:** Daniel D. Shaye, Iva Greenwald

There was an error in the Funding statement. The correct Funding is:

This work was funded by the Howard Hughes Medical Institute and also supported by National Institutes of Health grant CA095389 to IG. The funders had no role in study design, data collection and analysis, decision to publish, or preparation of the manuscript.

