# Supplementary material for: OrthoList: A Compendium of C. elegans Genes with Human Orthologs
Source: PLoS One. 2011 May 25;6(5):e20085. doi: 10.1371/journal.pone.0020085 (PMC3102077; doi:10.1371/journal.pone.0020085)
Supplement: Table S2 — OrthoList statistics. A) Number of unique hits found by each program. B) Congruence between programs (% hits shared). (DOC) [file pone.0020085.s002.doc]

**A) Uniques in OrthoList, by method**

| **Method** | **% (#) of total in**  **OrthoList (7,663 genes)** |
| --- | --- |
| Ensembl Compara | 11.7% (900) |
| OrthoMCL | 3.4% (258) |
| InParanoid | 6.4% (487) |
| HomoloGene | 0.9% (72) |
| Total | 22.4% (1717) |

**B) Congruence by method (% of hits in each method predicted by others)**

| **Method**  **(# of orthologs predicted)** | **%(#) predicted by three others.** | **% (#) predicted by two others** | **%(#) predicted by one other** | **%(#) predicted**  **by method alone** |
| --- | --- | --- | --- | --- |
| Ensembl Compara (6,467) | 52% (3,386) | 22% (1,395) | 12% (786) | 14% (900) |
| OrthoMCL (5,687) | 60% (3,386) | 25% (1,423) | 11% (620) | 5% (258) |
| InParanoid (5,619) | 60% (3,386) | 23% (1,299) | 8% (447) | 9% (487) |
| HomoloGene (4,141) | 82% (3,386) | 12% (482) | 5% (201) | 2% (72) |
